# Activation of Young LINE-1 Elements by CRISPRa

**DOI:** 10.3390/ijms25010424

**Published:** 2023-12-28

**Authors:** Bei Tong, Yuhua Sun

**Affiliations:** 1 Key Laboratory of Breeding Biotechnology and Sustainable Aquaculture, Institute of Hydrobiology, Chinese Academy of Sciences, Wuhan 430072, China; 2 The Innovation of Seed Design, Chinese Academy of Sciences, Wuhan 430072, China; 3 Hubei Hongshan Laboratory, Wuhan 430070, China

**Keywords:** LINE-1, CRISPR, disease, cancer

## Abstract

Long interspersed element-1 (LINE-1; L1s) are mobile genetic elements that comprise nearly 20% of the human genome. L1s have been shown to have important functions in various biological processes, and their dysfunction is thought to be linked with diseases and cancers. However, the roles of the repetitive elements are largely not understood. While the CRISPR activation (CRISPRa) system based on catalytically deadCas9 (dCas9) is widely used for genome-wide interrogation of gene function and genetic interaction, few studies have been conducted on L1s. Here, we report using the CRISPRa method to efficiently activate L1s in human L02 cells, a derivative of the HeLa cancer cell line. After CRISPRa, the young L1 subfamilies such as L1HS/L1PA1 and L1PA2 are found to be expressed at higher levels than the older L1s. The L1s with high levels of transcription are closer to full-length and are more densely occupied by the YY1 transcription factor. The activated L1s can either be mis-spliced to form chimeric transcripts or act as alternative promoters or enhancers to facilitate the expression of neighboring genes. The method described here can be used for studying the functional roles of young L1s in cultured cells of interest.

## 1. Introduction

Approximately 42% of the human genome is composed of repetitive elements (REs), such as long interspersed element-1 (LINE-1), endogenous retroviruses (ERVs), and short interspersed elements (SINE) [1]. L1s are abundant retrotransposons that comprise about 17% of the human genome. While around 500,000 L1 sequences exist in the human genome, the majority of them are non-functional due to 5′ truncations, deletions, and inversions [2]. Most of these sequences belong to ancestral/old LINE-1 subfamilies, such as L1M (mammalian-specific, oldest), no longer mobilize, and are thought to be largely non-functional. Only a small fraction of LINE-1 sequences, including L1HS/L1PA1 (human-specific, youngest) and the young L1PA subfamilies from L1P (primate-specific, intermediate), are of full length and capable of retrotransposition [3]. A human full-length L1 transcript is 6 kb long and has a 900 nt 5′-untranslated region (UTR) that functions as a RNA polymerase II internal promoter, two open reading frames (ORF1 and ORF2), a short 3′-UTR, and a poly(A) tail. 

Contrary to the earlier notion of them being ‘junk DNA’, recent studies have demonstrated the significant functional roles of REs like LINE-1 and ERVs in various biological processes [4,5]. For instance, LINE-1 RNA can act as a nuclear scaffold for recruiting Nucleolin/KAP1, repressing the 2C transcripts, regulating RNA synthesis and splicing, and remodeling the chromatin state [6,7,8]. Functionally, LINE1 is required for ESC self-renewal and embryonic development and plays an important role in regulating T cell quiescence and exhaustion [9]. However, aberrant activation of L1s is harmful to the stability of the human genome and is thought to be closely linked to various diseases, including neurodevelopmental disorders [10,11,12]. Although the etiology remains unclear, studies have suggested that activation of the repetitive elements may increase the risk of diseases by triggering an abnormal immune/inflammatory response and/or by altering the expression of nearby genes [13,14]. 

The host genome has evolved many mechanisms to restrict L1 activity at the transcriptional level, including epigenetic regulation such as DNA methylation and repressive histone modifications [15]. In cancer and disease conditions, however, many of the epigenetic mechanisms are disrupted, resulting in the activation and mobilization of the L1s [13]. For instance, mutation in *Mecp2* (*methyl-CpG binding protein 2*), which encodes a methyl-DNA-binding protein, leads to Rett syndrome and autism spectrum disorders with increased expression of L1s [4]. Moreover, environmental factors such as pollutants can cause abnormal expression of L1s by altering its DNA methylation and histone modifications [16,17]. 

Our current understanding of L1 function has been primarily based on loss-of-function studies, and gain-of-function studies have been scarce [18]. By fusing transcription activators with dCas9, gene activation can be achieved by the introduction of single-guide RNAs (sgRNAs) [19,20]. Here, we applied the CRISPRa method to study the activation of L1 elements in L02 cells. After CRISPRa, the young L1 subfamilies are found to be expressed at higher levels than the older L1s. The L1s with high levels of transcription are closer to full-length and are more densely occupied by the YY1 transcription factor. The activated L1s can either be mis-spliced to form chimeric transcripts or act as alternative promoters or enhancers to facilitate the expression of neighboring genes. 

## 2. Results

### 2.1. Efficient Activation of Young LINE-1 by CRISPRa

**Figure 1:**
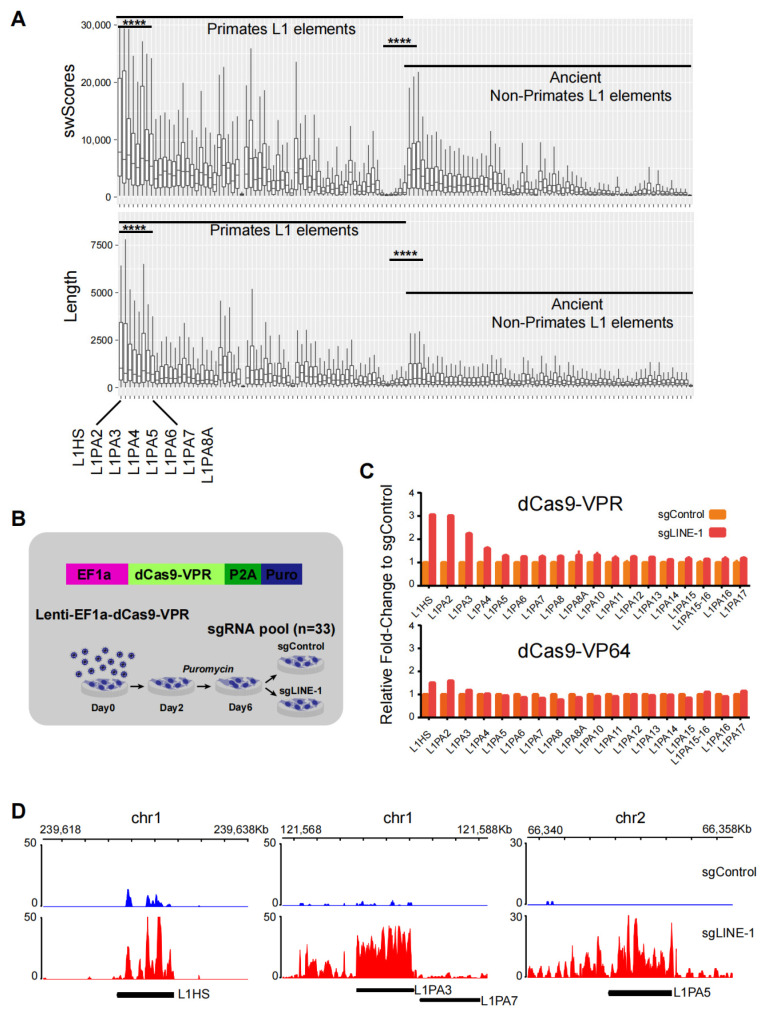
Activation of LINE-1 elements by CRISPRa in L02 cells. (**A**) Distribution of swScores and lengths of LINE-1 elements between primates and non-primates. ****: *p*< 0.0001. (**B**) Diagram illustrating the CRISPRa strategy for LINE-1 elements. (**C**) Bar graph showing the relative expression levels of the indicated LINE-1 subfamilies, based on RNA-seq results. Up: the dCas9-VPR method; bottom: the dCas9-VP64 method. (**D**) Genomic snapshots showing the up-regulation of the indicated L1s.

We downloaded the data from the UCSC (University of California, Santa Cruz, CA, USA) repeatmasker and analyzed LINE L1 elements from primates and ancient non-primates. At a global level, L1s were more conserved in primates than in ancient non-primates, as revealed by the Smith-Waterman dynamic alignment score (swScores). L1s with high swScores have more similar DNA sequences to the reference DNA sequence of the repetitive elements in the Dfam (http://www.dfam.org/ (accessed on 6 July 2022)) database. And the distribution of primate L1 lengths had a significantly larger mean than that of non-primate L1 lengths (*p* < 0.0001) (Figure 1A). In primates, the evolutionarily young L1s, such as the L1HS and other L1PA subfamilies (L1PA2-8), were ranked among the top 10 in terms of sequence length. As these young L1 elements are known to be potentially capable of transposing autonomously or retrotransposing competently [12], we focused on them in this study.

To activate the young L1s, we employed the CRISPRa method in L02 cells. CRISPRa exploits the LINE-1-specific gRNA pool and catalytically dead Cas9 (dCas9) fused to transcription activator VP64 or VPR for programmable L1 activation (Figure 1B; Table S1). The RNA-sequencing (RNA-seq) was used to evaluate the effects of L1 activation. The RNA-seq results showed that L1s can be activated in both the dCas9-VPR and dCas9-VP64 L02 cell lines, with the young L1 subfamilies such as L1HS/L1PA1 and L1PA2 being expressed at higher levels than the older L1s. In general, however, L1s were found to be more highly expressed in the dCas9-VPR cell line than in the dCas9-VP64 line (Figure 1C). The snapshots for the selected L1 loci were shown as representatives (Figure 1D). In the subsequent studies, we primarily analyzed the RNA-seq data from the dCas9-VPR line (Figure S1). 

### 2.2. Activation of nearby Genes by LINE-1 Elements

**Figure 2:**
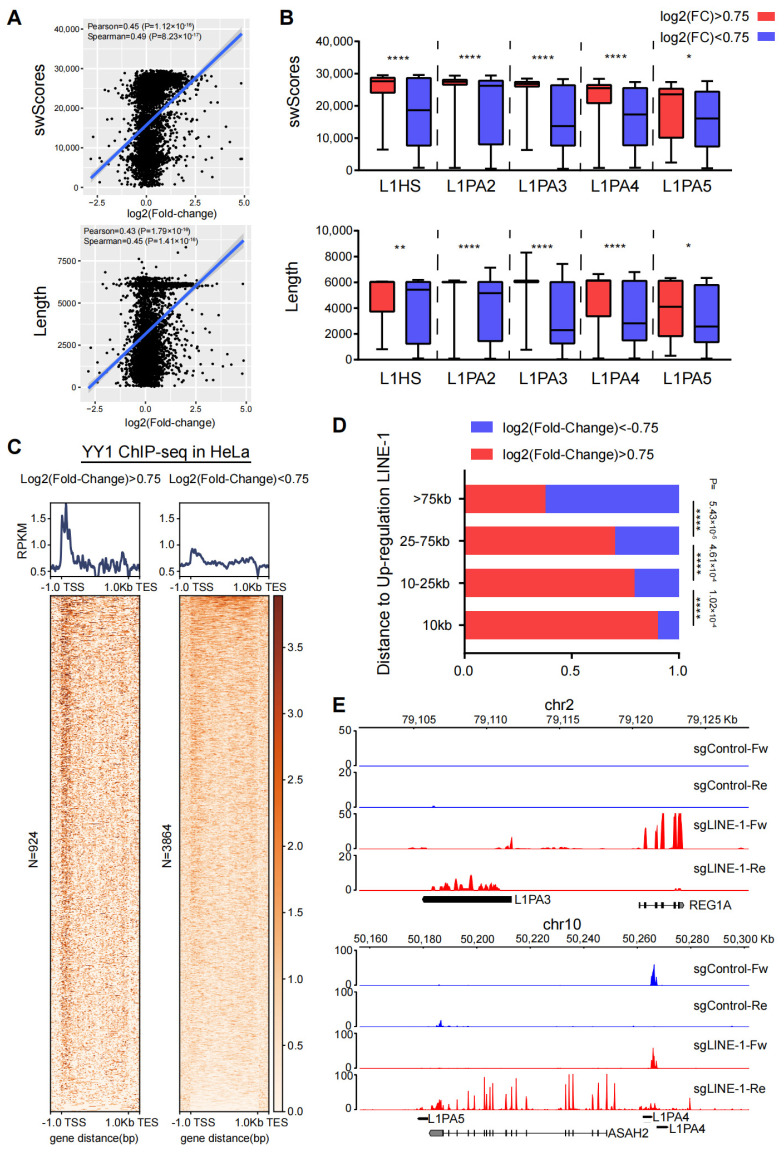
Activation of LINE-1 is associated with up-regulation of nearby genes. (**A**) Dot plot showing the correlation between expression of activated L1 elements and the swScores (up panel) and between expression of activated L1 elements and their length (bottom). Pearson: Pearson correlation; Spearman: Spearman correlation. (**B**) Box plot showing the distribution of swScores (up) and length (bottom) between highly expressed (red) and lowly expressed (blue) L1s. *: *p* < 0.05, **: *p* < 0.01, ****: *p* < 0.0001, (**C**) Heat map showing YY1 ChIP-seq signals at L1s with high (left) and low (right) levels of transcription in HeLa cells. (**D**) Distance distribution between the activated L1 elements and the transcription start sites of up-regulated genes. *X*-axis: proportion of up-regulated genes in the highly and lowly expressed L1s. *Y*-axis: the distance to the closest L1s. The P-values were shown on the right. (**E**) Snapshots showing expression levels of the indicated L1 elements and the nearby genes. The genome assembly version was hg38, and PyGenome Tracks was used to make the browser snapshots [23]. The bigwig files were generated by deeptools [24], and the FPKM (Fragments Per Kilobase of exon model per Million mapped fragments) was used to normalize the bigwig files. Fw: forward; Re: reverse.

Based on the scatter plot analysis, L1 elements that have high swScores or are long had a range of expression increases when activated by CRISPRa, while L1 elements that have low swScores or are short tended to not have expression increases (Figure 2A). For a more comprehensive investigation, each L1 subfamily was divided into a highly expressed group (log2 fold change *>* 0.75) and a lowly expressed group (log2 fold change < 0.75), based on the expression levels (Figure 2B). In general, the highly expressed L1s tended to be closer to full-length than the lowly expressed ones. 

YY1 has been shown to be required for human LINE-1 transcription initiation by directly binding to its promoter [21]. We downloaded YY1 ChIP-seq (chromatin immunoprecipitation followed by sequencing) data that were generated in HeLa cells [22]. Analysis of YY1 ChIP data showed that the 5′ untranslated regions (UTR) of the young L1s with high levels of transcription are more densely occupied by the YY1 transcription factor (Figure 2C), which indicated that greater YY1 enrichment at the 5′ UTR was correlated with higher expression of L1s (Figure S2).

Previous studies have shown that repetitive elements can contribute to regulatory activities as alternative promoters or enhancers [14]. We grouped genes based on their genomic distance from L1s and asked whether activation of L1s led to a change in the expression of neighboring genes. We divided L1s into two groups based on the expression levels after CRISPRa. In the highly expressed L1 group, genes located closer to the L1s were prone to being activated and exhibited significantly higher expression levels than genes far from the L1s, while in the lowly expressed L1 group, this was not the case (Figure 2D,E). The observation supported the idea that activated L1s can function as alternative promoters or enhancers to facilitate the expression of neighboring genes. 

### 2.3. Modulation of nearby Genes by Sense and Anti-Sense Transcripts of L1s 

**Figure 3:**
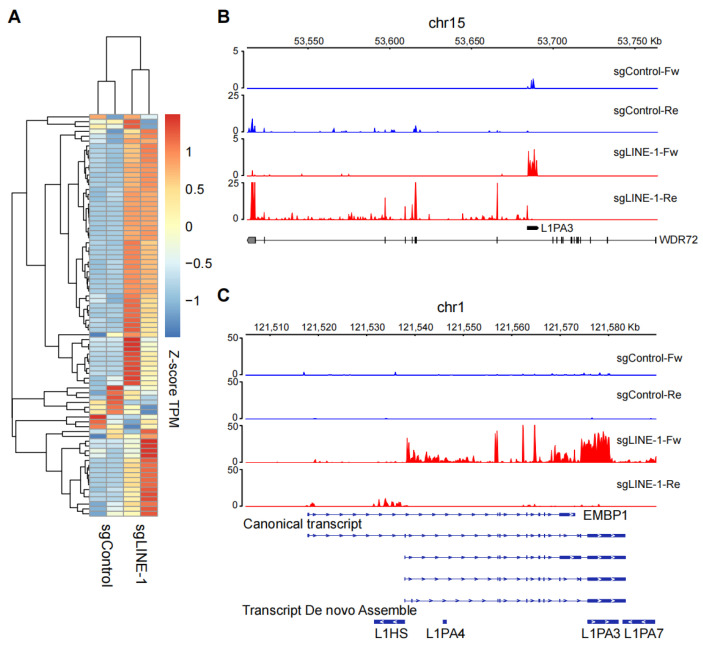
Fusion transcripts are formed between L1 and the nearby genes. (**A**) Heat map showing the expression of the de novo assembled L1 fusion transcripts, based on two replicates of CRISPRa experiments. (**B**) Genomic views of RNA-seq signals showing the expression of the *WDR72-L1* fusion gene. Fw: forward; Re: reverse. (**C**) Genomic views of RNA-seq signals showing the expression of the *EMBP1-L1* fusion gene.

We went on to investigate in more detail how activation of L1s leads to deregulation of nearby genes. We analyzed the expression of the de novo assembled L1 fusion transcripts, chimeric mRNAs encoded from the joined parts of a gene, and a L1 sequence. After CRISPRa, the majority of fusion genes were up-regulated (Figure 3A). L1s can be transcribed in the sense or anti-sense direction, depending on the genomic localization (Figure 3B,C). We found that both the sense and anti-sense sequences of L1s may play roles in modulating gene expression. For instance, the sense sequence of L1PA3 near the *WDR72* gene can function as an alternative promoter to facilitate the expression of *WDR72* from the anti-sense strand (Figure 3B), and the anti-sense product of L1PA3 may function as an enhancer to promote REG1A expression from the sense strand (Figure 2E). Interestingly, we observed that a gene’s expression can be influenced by the activation of elements from multiple L1 subfamilies. For instance, a sense L1PA3 sequence downstream of the *EMBP1* gene can be mis-spliced to form chimeric transcripts (Figure 3C), and an anti-sense of the intronic L1HS may function as an alternative promoter to facilitate their expression. 

### 2.4. Identification of Differentially Expressed Genes by L1 Activation 

**Figure 4:**
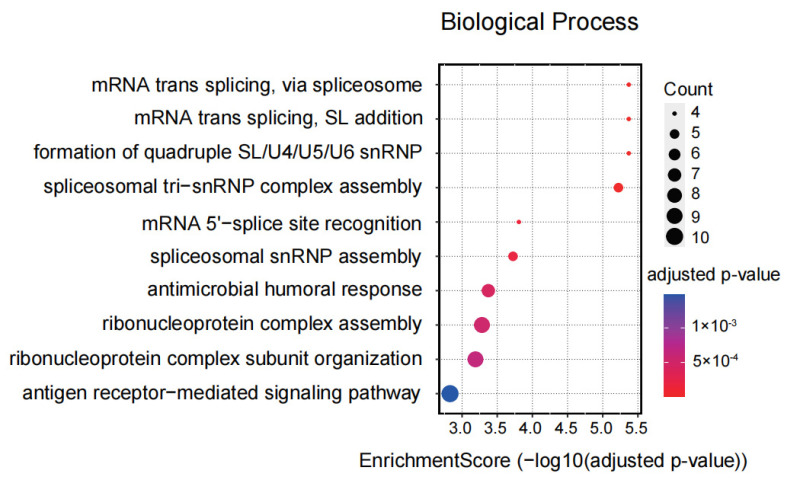
The GO terms of DEGs between control- and CRISPRa dCas9-VPR cells.

A total of 1182 differentially expressed genes (DEGs) were identified between control- and CRISPRa dCas9-VPR L02 cells (*p* < 0.05; log2(fold-change) > 0.75). To explore the functional properties of DEGs and infer the underlying mechanisms by L1 activation, we performed the Gene Ontology (GO) analysis. The results showed enriched GO terms such as antimicrobial humoral response, RNA splicing, T-cell receptor complex, and antigen-receptor-mediated signaling pathway (Figure 4). 

### 2.5. L1s Are Expressed in a Human Tissue Specific Manner

**Figure 5:**
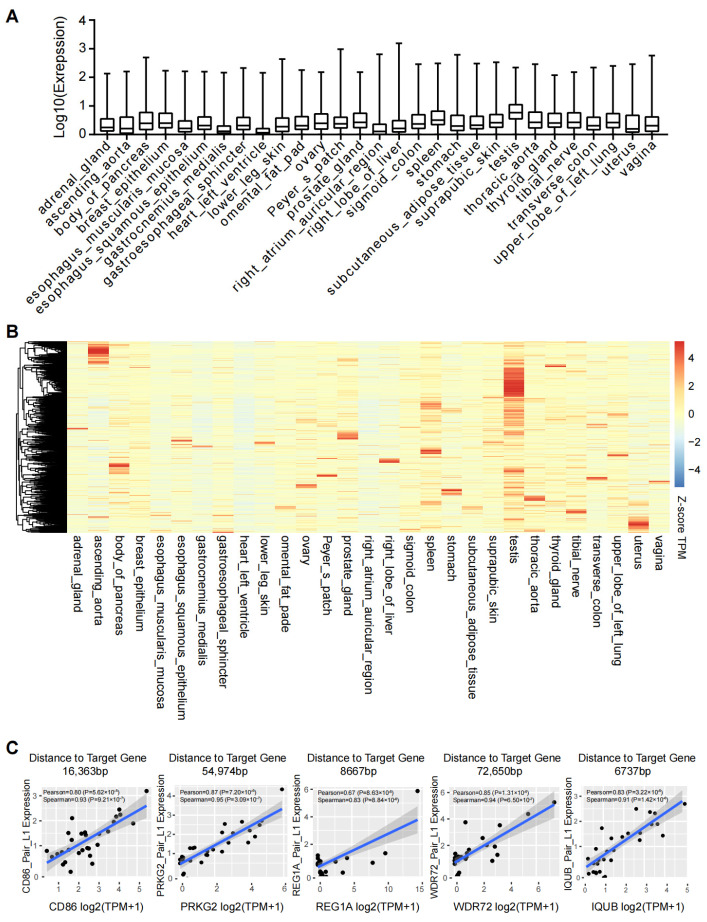
Tissue-specific expression of L1 elements in humans. (**A**) Boxplots showing the overall quantification of L1 expression levels in the indicated human tissues and organs, based on the ENCODE database. (**B**) Heat map showing the expression of individual L1 elements in the indicated human tissues and organs. (**C**) Scatter plot showing the correlation between the indicated gene and L1 expression in 29 normal human tissues. Each black dot represents a tissue. The distance of each L1 locus to the indicated gene was shown. Pearson: Pearson correlation; Spearman: Spearman correlation.

To understand the L1 function in normal physiological conditions, we analyzed L1 expression from 29 human tissues and organs. The results of quantification showed that the overall expression levels of L1s varied among different tissues or organs, with the highest expression observed in the testis (Figure 5A). The expression of L1s displayed a tissue-specific pattern (Figure 5B). For instance, L1HS was abundantly expressed in the thyroid_gland and prostate_gland, L1PA2 was highly enriched in the thoracic aorta, and L1PA3 was more expressed in the pancreas, as shown in the heat map. These observations were in line with the fact that L1s are transcribed in a cell-type-specific manner and that tissue-specific active promoters are enriched with the L1 family of transposons [25]. Nevertheless, certain L1s are co-expressed with protein-coding genes in multiple human tissues (Figure 5C), which implies that they may have general functions.

## 3. Discussion

Human neural disorders and cancers are closely linked with abnormal activation of L1s [4,13,26]. In this work, we used a simple method to efficiently activate a subset of L1s to study its function in cultured cells. We found that evolutionarily young L1s such as L1HS and L1PA2 were expressed at higher levels than older L1s after the CRISPRa. The reason behind this remains unclear, but it may be linked with the genomic and/or epigenomic features of the young L1s. Previous studies have shown that the young L1s are decorated with specific sets of histone marks, highly marked by RNA m6A modification, occupied by chromatin regulators such as Morc2 and the human silencing complex (HUSH), generally confined to deep intronic regions, and contain highly multivalent binding sites [6,7,8,9,10,11,12,27]. In fact, these young L1s have been shown to be more sensitive to epigenetic changes than older L1s. For instance, the young L1s in neural progenitors are sensitive to DNA methylation and are specifically activated upon DNMT1 depletion [8,10]. In ESCs, the young L1s, which are marked by m^6^A, are de-repressed after removal of SETDB1-mediated H3K9me3 [28]. Another interesting observation was that the young L1s with high levels of transcription by CRISPRa are more densely occupied by the YY1 transcription factor (TF) than their counterparts with low levels of transcription. Further functional analysis of YY1 in the transcriptional regulation of the young L1s is being conducted in the lab.

**Figure 6:**
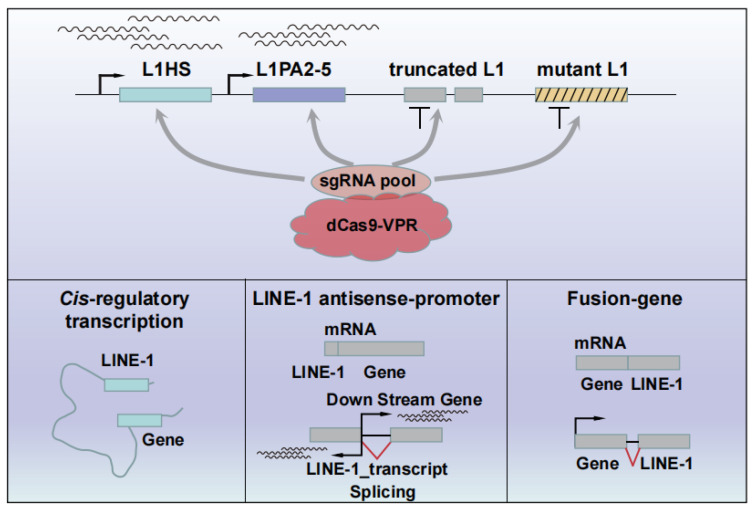
Cartoon depicting the roles of L1s in the regulation of gene expression. LINE-1 elements can function as enhancers, promoters, and parts of the chimeric transcripts, whereas the truncated and mutated L1s lack these activities.

Previous studies have shown that L1s may disrupt protein-coding genes, act as alternative promoters, function as long non-coding RNAs, and be spliced into mRNAs with nearby gene products [8,12]. How these pathways downstream of L1 activation converge on gene expression has been scarcely reported. Here, we showed that L1s can either be mis-spliced to form chimeric transcripts or act as alternative promoters to affect the expression of neighboring genes (Figure 6). Interestingly, we additionally found that a gene’s expression can be influenced by the activation of elements from multiple L1 subfamilies. For instance, a sense L1PA3 sequence downstream of the *EMBP1* gene may be mis-spliced to form chimeric transcripts, and an anti-sense of the intronic L1HS may function as an alternative promoter or enhancer to facilitate their expression. In the future, it will be interesting to investigate whether this is the general regulation mechanism.

By performing the GO analysis, we have studied the potential functional roles of young L1 activation in L02 cells. The results highlighted biological events such as the antimicrobial humoral response, RNA splicing via the spliceosome, and the immune response. These were in line with recent reports showing that L1 activation can lead to tumor or neuronal diseases by activating immune-related pathways [9,13] and that young L1s recruit RNA-binding proteins to regulate RNA splicing and contribute to lineage-specific transcripts [6]. Thus, our work demonstrates that gain-of-function L1 studies by CRISPRa can provide important information regarding the pathological effects of abnormal L1 activation. 

This work has some limitations. First, the CRISPRa experiments were performed using the L02 cell line, a derivative of the HeLa cell line. The genome of this cancerous cell line might have been substantially rearranged, making information about the proximity of genes to L1 elements unreliable, and results from activating regions in these cells may be different from those in healthy cells. Second, as the guides were tested in a single gRNA pool, the effects of individual guides and specific combinations of guides could not be determined. Third, the effects of activation of a specific young L1 subfamily are not clear. Despite the limitations, we show that the young L1s can be efficiently activated by CRISPRa, resulting in a global change in gene expression. The method described here can be used for studying the functional roles of L1s in cultured cells of interest in the future. For instance, one can activate a specific subfamily of L1s in neural cells to study the downstream cellular and molecular events that may cause neuronal dysfunction.

## 4. Materials and Methods

### 4.1. Cell Culture

The human L02 cell line (#TCH-C446) was purchased from Shanghai Cell Bank. The L02 and the dCas9-VPR LINE-1 L02 cell lines (see below) were cultured in DMEM (Dulbecco’s Modification of Eagle’s Medium) supplemented with 10% FBS (TransGen Biotech, #FS301-02, Beijing, China), 1% L-Glutamine, and 1% Penicillin-Streptomycin. L02 cells were passaged every 4 days with 0.25% trypsin-EDTA (Ethylene Diamine Tetraacetie Acid) (#T4049, Sigma, Saint Louis, MI, USA). After passaging, 2 × 10^5^ cells were seeded in 6 well plates.

HEK293T cells were used to make lentiviral particles. When HEK293T cells were cultured, the medium was DMEM containing 10% FBS, 1% L-Glutamine, and 1% Penicillin-Streptomycin. The list of reagents can be found in Supplementary Table S2.

### 4.2. Plasmids for the CRISPRa System

To manipulate the endogenous activity of L1s, the epigenome editing technique was used, based on the clustered regularly interspaced short palindromic repeats (CRISPR) system. Specifically, we used a CRISPR activation (CRISPRa) system by a strong tripartite transcriptional activator comprising VPR (VP64, P65, and Rta) [29], or VP64 activator. By fusing the VPR and VP64 activators with deactivated Cas9 (dCas9), locus-specific control of endogenous L1 expression can be achieved by the introduction of guide RNAs (gRNAs). The plasmids used, such as lenti-EF1a-dCas9-VPR-Puro (#99373) and lenti-EF1a-dCas9-VP64-Puro (#99371), were purchased from the Addgene company (Cambridge, MA, USA).

### 4.3. sgRNA Design

We designed 33 sgRNAs that are specifically targeted to the 5′ UTR of the L1HS and the indicated L1PA subfamilies. A total of 16,635 L1 loci were predicted to be targeted, covering all L1 subfamilies. The sgRNAs were designed using the MIT guide RNA design tool (www.CRISPR.MIT.edu (accessed on 6 April 2021)) or http://crispor.tefor.net/ (accessed on 6 April 2021). Guide RNA design and the sequences can be found in Supplementary Tables S1, S3 and S4. 

### 4.4. Lentiviral Production in HEK293T Cells

The method for lentiviral production has been described previously [30], which was modified according to the technical manual from Thermo Scientific (Waltham, MA, USA) Trans-Lentiviral Packaging kit (TLP4614). Briefly, 5 × 10^6^ cells were seeded on 10 cm plates and incubated overnight at 37 °C with 5% CO2. The next day, after changing the medium, 20 μg of lenti-EF1a-dCas9-VPR-Puro (Addgene, #99373, Cambridge, MA, USA), lentiviral packaging plasmids of 10 μg of psPAX2 (Addgene, #12260, Cambridge, MA, USA), and 10 μg of pMD2.G (Addgene, #12259, Cambridge, MA, USA) were transfected into HEK293T cells using a 120 μL PEI transfection reagent (#HY-K2014, MedChemExpress, Monmouth Junction, NJ, USA). After 72 h of incubation, the supernatants containing the lentivirus were collected. The supernatants containing the lentiviral particles were concentrated by using the Lenti-X concentrator reagents (#631231, Takara Bio, Dalian, China), according to the kit manual. After concentration, 25 μL of the lentiviral solution was loaded into 1.5 mL EP (Eppendorf, Hamburg, Germany) tubes and stored at −80 °C for later use. The titer of the concentrated lentiviral particles was determined by qRT-PCR analysis using the titration kit (#631235, Takara Bio, Dalian, China). 

### 4.5. LINE-1 Activation in L02 Cell Lines

LINE-1 was activated in L02 cells by a two-step strategy. First, the dCas9-VPR cell line was established by transduction of lentivirus into L02 cells. Briefly, 2 × 10^5^ L02 cells were seeded on 35 mm dishes and incubated overnight at 37 °C with 5% CO_2_. The next day, 25 μL of concentrated lentivirus were thawed at room temperature and used to infect the L02 cells at a MOI (multiplicity of infection) of 2. 24 h after transduction, the infected cells were selected with 2 μg/mL puromycin a week also generate the stable dCas9-VPR cell line. The dCas9-VP64 L02 cell line was established by the same protocol.

Next, the stable dCas9-VPR and dCas9-VP64 L02 cell lines were transfected with a sgRNA pool. Briefly, 2 × 10^5^ of the dCas9-VPR or dCas9-VP64 L02 cells were seeded on 35 mm dishes and incubated overnight at 37 °C with 5% CO_2_. The next day, a 40 μg sgRNA pool, including 33 sgRNAs targeting L1HS and L1PA subfamilies, was transfected into cells using the PEI transfection reagent. A total of 40 μg scramble sgRNAs were used as controls. In addition, 72 h after transfection, total RNA was extracted for further analysis (below).

### 4.6. Next-Generation Sequencing and Bioinformatics

Total RNA was extracted by the TRIzol reagent (#15596026, Thermo Scientific, Waltham, MA, USA). After removing the rRNA, a cDNA library was prepared and sequenced on Illumina Hiseq 2000 machines by the BGI company (Shenzhen, China).

The RNA sequencing reads were mapped to the Homo sapiens hg38 reference assembly using Hisat2 [31]. The gene annotation Genecode v29 was downloaded from “https://www.gencodegenes.org/ (accessed on 11 August 2022)” and raw counts were performed by FeatureCounts [32]. Then, raw counts of all genes were normalized by TPM (transcript per million). Differential expression analysis was performed by DEseq2 [32]. Genes with abs|log2(fold-change) > 0.75 and P-adj < 0.05 were considered differentially expressed genes (DEGs). RNA-seq data were aligned to the hg38 genome by Hisat2. The L1 reads were randomly assigned once. The de novo transcript assembly was performed by Stringtie [31], with a parameter of: -m 500 -a 20. 

### 4.7. ChIP-Seq Analysis

YY1 ChIP-seq data generated in HeLa cells by Zhang et al. [22] were downloaded from the ENCODE Project database (https://www.encodeproject.org/ (accessed on 12 November 2023)). ChIP-seq data were analyzed using the default parameters, except for bowtie2, where we used the parameters: “ -p 64 --very-sensitive --end-to-end --no-unal”. Briefly, raw data were filtered by trim_galore (https://www.bioinformatics.babraham.ac.uk/projects/trim_galore/ (accessed on 12 November 2023)) to generate the clean data with the parameter:-q 30, and the reads were then aligned to the human genome (hg38) using bowtie2 with the parameters: -p 64 --very-sensitive --end-to-end --no-unal [33]. Reads mapped to the mitochondrial genome were removed using samtools [34]. Only the best alignments were kept, and multi-mapped reads were randomly retained once. PCR duplicates were removed using Picard MarkDuplicates (http://broadinstitute.github.io/picard/ (accessed on 23 November 2023)). The bam alignment files of the same sample were merged by the samtools merge function. The merge bam files were transformed into normalized RPKM (Reads Per Kilobase per Million mapped reads) bigwig files. 

The annotation of hg38 LINE-1 elements was obtained from the UCSC Genome Browser RepeatMasker. For LINE-1 enrichment analysis, we calculated the total counts of each LINE-1 subfamily in the annotations and then normalized to RPKM (Reads Per Kilobase Million). The coverage signals in LINE-1 elements were generated by deeptools [24]. For individual LINE-1 elements, visual track views were generated based on uniquely mapped reads.

### 4.8. Gene Ontology Analysis

The differentially expressed genes (DEGs) were identified between control- and CRISPRa dCas9-VPR L02 cells. The GO analysis was performed at https://geneontology.org (accessed on 23-November-2023). The software information can be found here https://geneontology.org/docs/go-citation-policy/ (accessed on 23-November-2023).

### 4.9. Statistical Analysis

Statistical analysis was performed using the GraphPad Prism 8.0 software (* *p* < 0.05; ** *p* < 0.01; *** *p* < 0.001; **** *p* < 0.0001. n.s., not significant) with a two-tailed, unpaired Student’s *t*-test.

## Data Availability

All information is shown in the Section 4.

## Supplementary Materials

The supporting information can be downloaded at: https://www.mdpi.com/article/10.3390/ijms25010424/s1.

## Abbreviations

**Short**	**Full Name**
ASAH2	Neutral ceramidase
CTCF	CCCTC-binding factor
ChIP-seq	Chromatin Immunoprecipitation-sequencing
CRISPR	clustered regularly interspaced short palindromic repeat
CRISPRa	CRISPR activation
dCas9	dead CRISPR-associated endonuclease Cas9
DEGs	differentially expressed genes
DNMT1	DNA (cytosine-5)-methyltransferase 1
EMBP1	DNA-binding protein
ERVs	endogenous retrovirus
ESC	Embryonic stem cell
ENCODE	The Encylopedia of DNA Elements
FL-L1s	full-length L1s
gRNAs	guide RNAs
GO	gene ontology
HUSH	The human silencing hub complex
hg38	human genome 38
KAP1	cAMP-dependent protein kinase type I-beta regulatory subunit
KEGG	Kyoto Gene and Genomic Encyclopedia
L1M	L1 mammalian-specific
L1P	L1 primate-specific
LINE-1	Long interspersed element-1
L1HS/L1PA1	L1 human specific
Morc2	ATPase MORC2
MOI	Multiplicity of infection
Mecp2	methyl-CpG binding protein 2
POGZ	Pogo transposable element with ZNF domain
REs	repetitive elements
REG1A	Lithostathine-1-alpha
RNA-seq	RNA-sequencing
RPKM	Reads Per Kilobase per Million
swScore	Smith-Waterman Score
SETDB1	Histone-lysine N-methyltransferase SETDB1
SINE	short interspersed element
TPM	Transcript per million
UTR	Untranslated Region
UCSC	University of California Santa Cruz
VP64	The tetramer of virus protein 16
VPR	VP64, P65, and Rta
WDR72	WD repeat-containing protein 72
YY1	Transcriptional repressor protein YY1
ZGA	Zygotic genome activation
