# Supplementary material for: Activation of Young LINE-1 Elements by CRISPRa
Source: Int J Mol Sci. 2023 Dec 28;25(1):424. doi: 10.3390/ijms25010424 (PMC10778729; doi:10.3390/ijms25010424)
Supplement: Supplementary file 1 [file ijms-25-00424-s001.zip › Supplementray Figures.pdf]

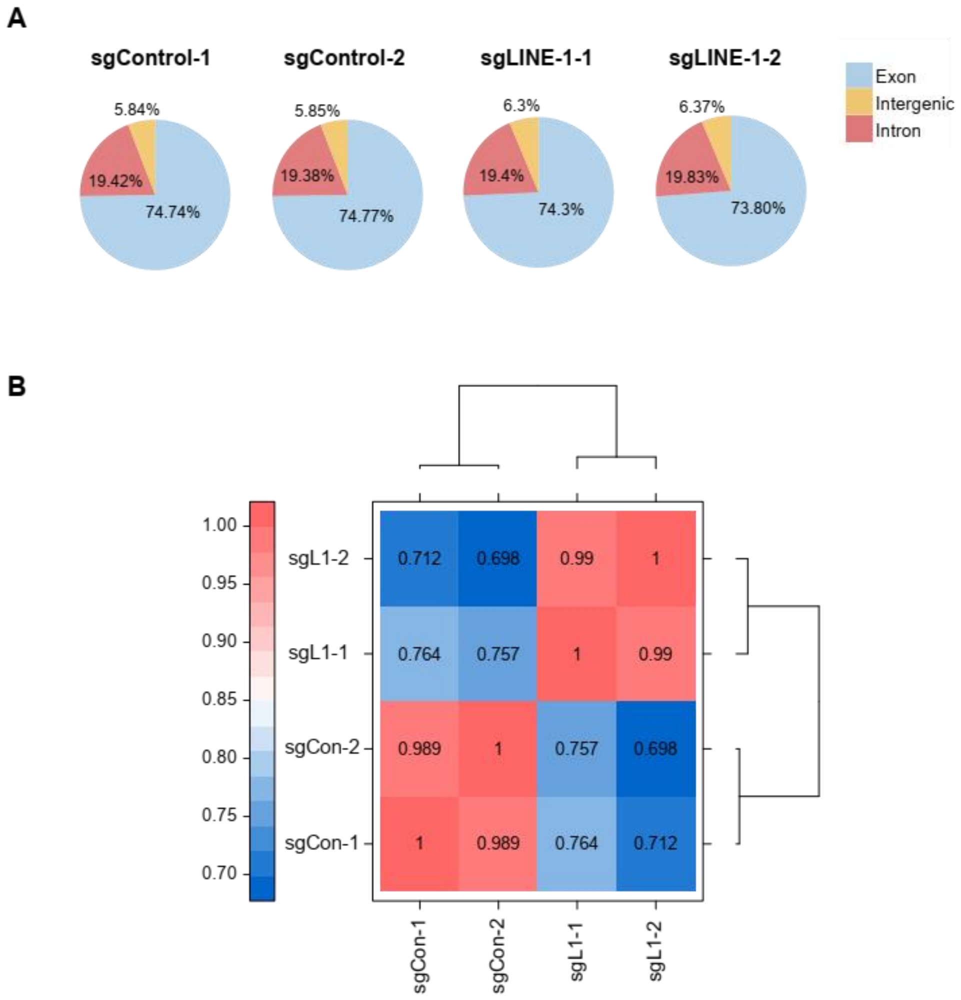

Supplementary Figure S1. Analysis of the CRISPRa data. (A) Pie chart displaying the genomic distribution of different reads from RNA-seq peaks. (B) Heat map representing Pearson correlations between control and dCas9-VP64 L02 cell lines, based on two replicates.

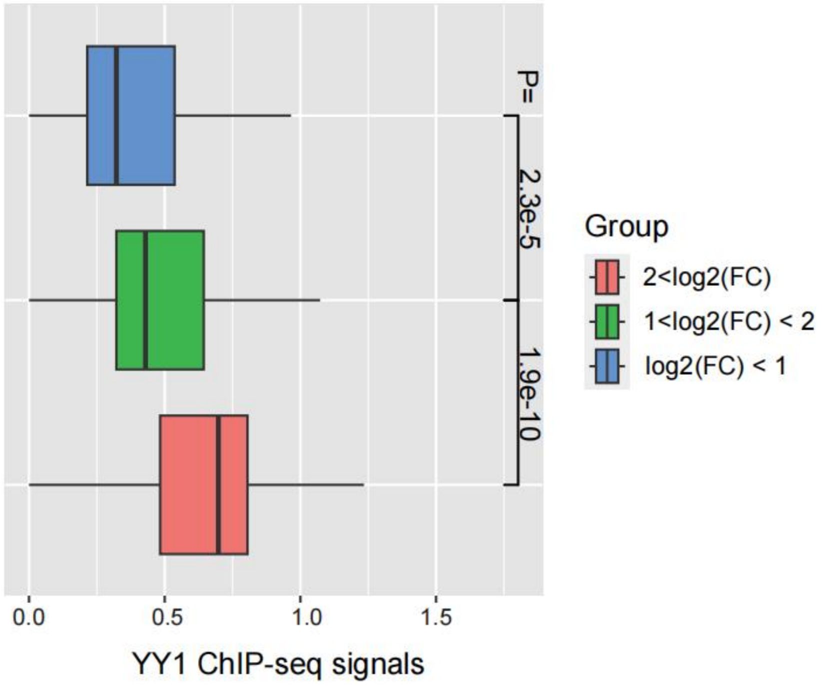

Supplementary Figure 2. The correlation between the YY1 ChIP-seq signals and the L1 expression. The young L1s after CRISPRa are divided into 3 groups: lowly expressed (log2 fold change< 1); medially expressed (1<log2 fold change< 2); and highly expressed (log2 fold change> 2).
